# Implementation and Evaluation of a Bystander Naloxone Training Course

**DOI:** 10.5811/westjem.60409

**Published:** 2024-04-09

**Authors:** Scott G. Weiner, Scott A. Goldberg, Cheryl Lang, Molly Jarman, Cory J. Miller, Sarah Li, Ewelina W. Stanek, Eric Goralnick

**Affiliations:** * Brigham and Women’s Hospital, Department of Emergency Medicine, Boston, Massachusetts; † Brigham and Women’s Hospital, Department of Surgery, Boston, Massachusetts

## Abstract

**Introduction:**

Bystander provision of naloxone is a key modality to reduce opioid overdose-related death. Naloxone training courses are available, but no standardized program exists. As part of a bystander empowerment course, we created and evaluated a brief naloxone training module.

**Methods:**

This was a retrospective evaluation of a naloxone training course, which was paired with Stop the Bleed training for hemorrhage control and was offered to administrative staff in an office building. Participants worked in an organization related to healthcare, but none were clinicians. The curriculum included the following topics: 1) background about the opioid epidemic; 2) how to recognize the signs of an opioid overdose; 3) actions not to take when encountering an overdose victim; 4) the correct steps to take when encountering an overdose victim; 5) an overview of naloxone products; and 6) Good Samaritan protection laws. The 20-minute didactic section was followed by a hands-on session with nasal naloxone kits and a simulation mannequin. The course was evaluated with the Opioid Overdose Knowledge (OOKS) and Opioid Overdose Attitudes (OOAS) scales for take-home naloxone training evaluation. We used the paired Wilcoxon signed-rank test to compare scores pre- and post-course.

**Results:**

Twenty-eight participants completed the course. The OOKS, measuring objective knowledge about opioid overdose and naloxone, had improved scores from a median of 73.2% (interquartile range [IQR] 68.3%–79.9%) to 91.5% (IQR 85.4%–95.1%), *P* < 0.001. The three domains on the OOAS score also showed statistically significant results. Competency to manage an overdose improved on a five-point scale from a median of 2.5 (IQR 2.4–2.9) to a median of 3.7 (IQR 3.5–4.1), *P* < 0.001. Concerns about managing an overdose decreased (improved) from a median of 2.3 (IQR 1.9–2.6) to median 1.8 (IQR 1.5–2.1), *P* < 0.001. Readiness to intervene in an opioid overdose improved from a median of 4 (IQR 3.8–4.2) to a median of 4.2 (IQR 4–4.2), *P* < 0.001.

**Conclusion:**

A brief course designed to teach bystanders about opioid overdose and naloxone was feasible and effective. We encourage hospitals and other organizations to use and promulgate this model. Furthermore, we suggest the convening of a national consortium to achieve consensus on program content and delivery.

## INTRODUCTION

Time is a critical contributing factor in patient outcomes in many emergencies. In the United States, the average response time by emergency medical services to a 9-1-1 call is seven minutes.^1^ To bridge this gap, many efforts have been launched to empower laypersons, who are typically first on the scene, to intervene and employ skills ranging from cardiopulmonary resuscitation (CPR) and automated external defibrillator (AED) use to bleeding control interventions.^2^ Basic Life Support (BLS) course content is based upon rigorous and frequently updated consensus (ie, American Heart Association [AHA] Guidelines Update for CPR and Emergency Cardiovascular Care).^3^^,^^4^ These courses are taught in a standardized fashion by the AHA and the American Red Cross. Likewise, the Stop the Bleed (STB) program, a national initiative launched in 2015 focused on empowering the public and public safety professionals to recognize and control life-threatening bleeding, has several types of courses, the most prominent being the American College of Surgeons’ (ACS) Basic Hemorrhage Control Course (BCon).^5^^,^^6^

While CPR, AED and STB training focus on preventable deaths, another significant source of preventable deaths is the opioid overdose epidemic, which remains one of the most pressing public health issues of our time, having claimed about 1,000,000 lives in the US since 1999.^7^ The number of overdose deaths has increased greatly in recent years, with yet another record number in 2021, predominantly due to fentanyl.^8^ Bystander naloxone administration, which can be used to reverse an opioid overdose, has been introduced as one potential mitigating factor. In 2018, the US Surgeon General issued an advisory on naloxone and opioid overdose that encourages community members who come into contact with people at risk for opioid overdose to know how to use naloxone and keep it within reach.^9^ Likewise, the US Department of Health and Human Services’ overdose prevention strategy includes harm reduction, with a goal to widen access to opioid overdose reversal treatments.^10^

Unlike CPR, there is no one standardized course for bystander naloxone training. Online courses are offered by agencies such as the Centers for Disease Control and Prevention (CDC),^11^ the American Red Cross,^12^ individual states (eg, Massachusetts^13^ and New York^14^), and other non-profits (eg, GetNaloxoneNow^15^). The courses lack a standardized core content, measures of effectiveness, or agreed-upon delivery methods (in person, hybrid, remote, simulation, didactic, etc). Although anecdotes exist of layperson use, we have a limited understanding of an effective, layperson naloxone-empowerment curriculum, and gaps remain in knowledge about training parameters and strategies.^16^

In this study, we evaluated an overdose-response naloxone training program administered to laypersons. We emphasized the structure and curriculum of the course and evaluated efficacy with a validated screening tool.

## METHODS

The naloxone course was designed to be a brief intervention with 20 minutes of didactics and 20 minutes of practical experience with a mannequin. The course was bundled with the ACS BCon course as part of a bystander empowerment program. Course instructors were three board-certified emergency physicians. The session took place at a professional office building. Although the participants worked in an organization related to healthcare, all worked as office staff and none were clinicians. Two identical sessions were offered, and both took place in June 2018 during normal business hours. Participants were not compensated specifically for participating but attended in lieu of their normal duties. We administered anonymous pre- and post-course evaluations. The project was determined to not meet the criteria for human subject research by the Mass General Brigham Human Research Office.

### Curriculum

Created by the course instructors, the curriculum included the following topics: 1) background about the opioid epidemic; 2) how to recognize the signs of an opioid overdose; 3) actions not to take when encountering an overdose victim; 4) the correct steps to take when encountering an overdose victim; 5) an overview of naloxone products; and 6) Good Samaritan protection laws. Content was created by first searching for existing training resources online, including training manuals from the states of New York (https://www.dhses.ny.gov/naloxone-information-first-responders) and Texas (https://txoti.org), and Canadian province Manitoba (https://www.gov.mb.ca/health/publichealth/docs/training_manual_overdose.pdf). This information was integrated with additional content from course instructor expertise into a didactic module containing 30 slides (Appendix 1), and participants were provided with a hard copy of the slides. The practical module entailed small groups around a simulation mannequin with a course instructor. Participants were able to practice with two types of naloxone kits (pre-packaged nasal naloxone spray and an autoinjector) on the mannequin. Discussion was encouraged until all participants’ questions and concerns were addressed.

### Course Evaluation

To evaluate the efficacy of the course, we used the Opioid Overdose Knowledge (OOKS) and Opioid Overdose Attitudes (OOAS) scales for take-home naloxone training evaluation.^17^ The first half of this validated tool (OOKS) asks objective questions about opioid overdose to evaluate trainee knowledge, including indicators of opioid overdose, how to manage an overdose, the mechanism of action of naloxone, and its duration of action. The second part (OOAS) asks questions pertaining to perceptions of competencies to manage an opioid overdose, concerns about managing an overdose, and readiness to intervene in an opioid overdose.

### Statistical Analysis

All participants completed pre- and post-evaluations on paper forms. Subjects were asked to write the same random four-digit number on each of the two evaluations for paired analysis purposes. Responses were transferred to a spreadsheet, and a second investigator confirmed the accuracy of the transcription. The OOKS scale is a series of true/false statements, and the correct answers were summed, with a total possible 41 points. We modified the original 45-point version slightly, as multiple points were possible for several individual questions (eg, “What is naloxone used for?” and “How can naloxone be administered?”) and we counted them only as one point each. There was also a choice of “don’t know” for several questions, and that was considered an incorrect answer as indicated in the scoring instructions. The OOAS scale is 28 questions divided into three domains and measured on a five-point Likert scale (5 = completely agree and 1 = completely disagree). Although the post-test OOKS results and one of the domains on the OOAS were normally distributed as determined by the Shapiro-Wilk test, the remainder of results were non-normal. Thus, all results, including the scales on each domain of the OOAS and the overall score on the OOKS, are described with medians and interquartile range (IQR) and compared with the paired Wilcoxon signed-rank test. We analyzed data with JMP v16 (JMP Statistical Discovery LLC, Cary. NC).

## RESULTS

Twenty-eight participants took the course. All completed the pre-test and the post-test, although three participants did not answer all questions on the pre-test OOAS scale. Therefore, the corresponding answers in the domains for these three individuals on the post-test were not included in the analysis. The OOKS, measuring objective knowledge about opioid overdose and naloxone, had improved scores from a median of 73.2% (IQR 68.3%–79.9%) to 91.5% (IQR 85.4%–95.1%), *P* < 0.001. The three domains on the OOAS score also showed statistically significant results. Competency to manage an overdose improved from a median of 2.5 (IQR 2.4–2.9) to a median of 3.7 (IQR 3.5–4.1), *P* < 0.001. Concerns about managing an overdose decreased (improved) from a median of 2.3 (IQR 1.9–2.6) to median 1.8 (IQR 1.5–2.1), *P* < 0.001. Readiness to intervene in an opioid overdose improved from a median of 4 (IQR 3.8–4.2) to a median of 4.2 (IQR 4–4.2), *P* < 0.001.

## DISCUSSION

In creating and evaluating a naloxone training program for bystanders, we found improvement in both subjective attitudes and objective knowledge about opioid overdose and naloxone. The training is relatively brief (lasting under an hour) and effective. We have subsequently taught this curriculum several times to local community organizations, including those who work with people who use drugs. Although we did not measure objective outcomes subsequently, the concept of bystander empowerment, teaching both naloxone and STB skills, has been well received and represents important outreach from our hospital to the local community.

One key question that remains is whether this training is necessary for bystanders. In our previous research, we found that 49 of 50 bystanders were able to correctly administer naloxone in a simulated experience on a public sidewalk with guidance by a simulated 911 dispatcher.^18^ However, not everyone will have the guidance of a dispatcher when using naloxone, and there may be confusion about how to use the kit and the timing of a second dose (if needed) without that assistance. Bystander training may also be valuable as a way to foster self-efficacy, increasing the likelihood that a layperson will recognize and respond to an overdose. In our course, we also cover when bystanders should administer naloxone and dispel myths about any harm that can be caused by giving it, as well as how to access naloxone.

Naloxone for bystanders is currently available via standing order in several states, meaning that individuals can obtain it from pharmacies without a prescription.^19^^–^^22^ Standing orders are associated with reductions in fatal overdoses in the community.^23^ The current packaging of prescription nasal naloxone has a flap that opens giving just-in-time (JIT) instructions to the bystander, but that may not be sufficient. The US Food and Drug Administration (FDA) recently approved making nasal naloxone an over-the-counter medication, even though its briefing document described several cases of incorrectly administered naloxone, including an individual who did not place the tip of the dispenser fully in the nostril, someone who squeezed the device but did not push the plunger, another who placed the device upside down so that the plunger was in the nostril, and several individuals who did not wait 2-3 minutes before administering a second dose.^24^ While the FDA advisors voted unanimously to make naloxone available without a prescription,^25^ these errors in administration indicate the need for a bystander course that could further improve outcomes.

Another reason to teach such a course is to address stigma, which is pervasive when considering opioid use disorder (OUD).^26^ A recent study of individuals who did not use illicit opioids themselves but knew others who did reported stigma about OUD and misinformation about opioid-related risks.^27^ Naloxone-based interventions can introduce the concept of harm reduction, empower bystanders, and encourage individuals to carry naloxone in case they encounter an overdose victim.^28^

Although not a part of our study, despite the positive results on our objective and subjective testing, we do encourage the creation of standardized training. The STB BCon portion of our course was created and endorsed by the ACS, using standardized content and certified trainers. A similar process could be used for naloxone, either as part of a BLS training, such as from the AHA or American Red Cross, from a specialty society, such as the American Academy of Emergency Medicine, the American College of Emergency Physicians, or the American Society of Addiction Medicine, or from a national advocacy group such as Shatterproof. Such branding and promotion may empower more bystanders to become trained and further reduce stigma and misconceptions about OUD among the general population.

While CPR training for laypersons is the gold standard, many gaps in implementing bystander training remain, and an investment in the study of the effectiveness of the relatively simple steps of naloxone administration may help us learn and improve techniques of CPR and STB training as well. For example, despite educational initiatives that began in the 20th century, only one-third of out-of-hospital cardiac arrest patients receive bystander CPR. Time, location, and duration have all been perceived by the public as barriers to CPR classes.^29^ Blacks and Hispanics are less likely than Whites to receive CPR at home or in public.^30^ In the last decade, there have been many initiatives with variable efficacy, in most cases not measured, to use JIT tools like flashcards, video or talking kits to provide users with real-time instructions for the use of automated external defibrillators or STB equipment. While the agreement of course content and identifying efficacy is a first step, future work should also focus on developing, trialing, and scaling effective JIT naloxone-administration tools.

## LIMITATIONS

There are limitations to our study. We taught this course to a small sample of administrative professionals in a suburb of Massachusetts, a state with a high burden of opioid-related overdose. It is possible that bystanders from different backgrounds and geographic locations would have answered the questions differently. We also did not collect any demographic data about our study participants to protect confidentiality. However, this information might have determined the characteristics of individuals who may benefit most from the training. The content of the practical session of the course was not standardized. Finally, we did not measure knowledge retention or use of naloxone following the course.

## CONCLUSION

A brief course designed to teach bystanders about opioid overdose and naloxone was feasible and effective. We encourage hospitals and other organizations to use and promulgate this model. Furthermore, we suggest convening of a national consortium to achieve consensus on program content, delivery, and opportunities for development of just-in-time tools to administer naloxone.

## Supplementary Information




