# Supplementary material for: Implementation and Evaluation of a Bystander Naloxone Training Course
Source: West J Emerg Med. 2024 Apr 9;25(3):320–4. doi: 10.5811/westjem.60409 (PMC11112654; doi:10.5811/westjem.60409)
Supplement: Supplementary file 1 [file wjem-25-320-s001.pdf]

Opioid Overdose Response and Naloxone Training

Department of Emergency Medicine

Brigham and Women's Hospital

Training Objectives

- Background about the opioid epidemic
- Recognize the signs of an opioid overdose and identify its causes and risks
- Describe what NOT to do during an opioid overdose
- Know the steps to follow when encountering an opioid overdose
- Understand administration of naloxone products, including “Good Samaritan” protection law

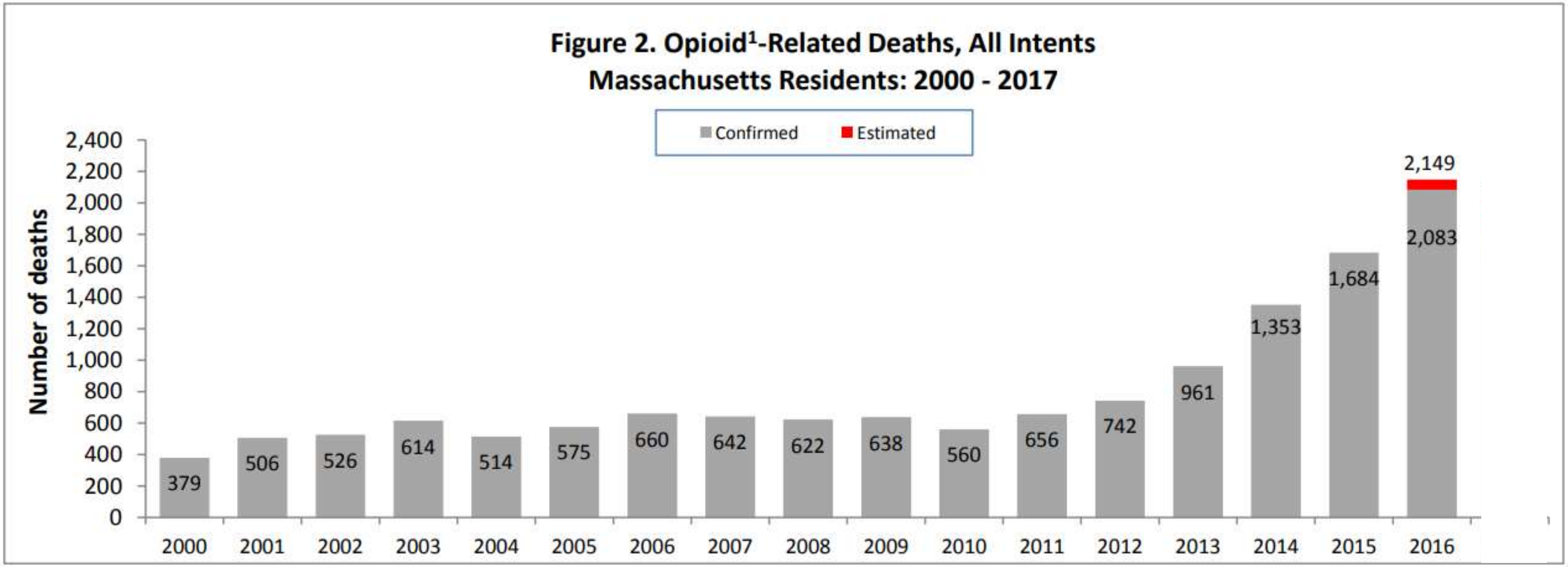

https://www.mass.gov/files/documents/2018/05/22/Opioid-related%20Overdose%20Deaths%20among%20MA%20Residents%20-%20May%202018.pdf

Fentanyl Epidemic

Figure 4. Age-adjusted drug overdose death rates, by opioid category: United States, 1999–2016

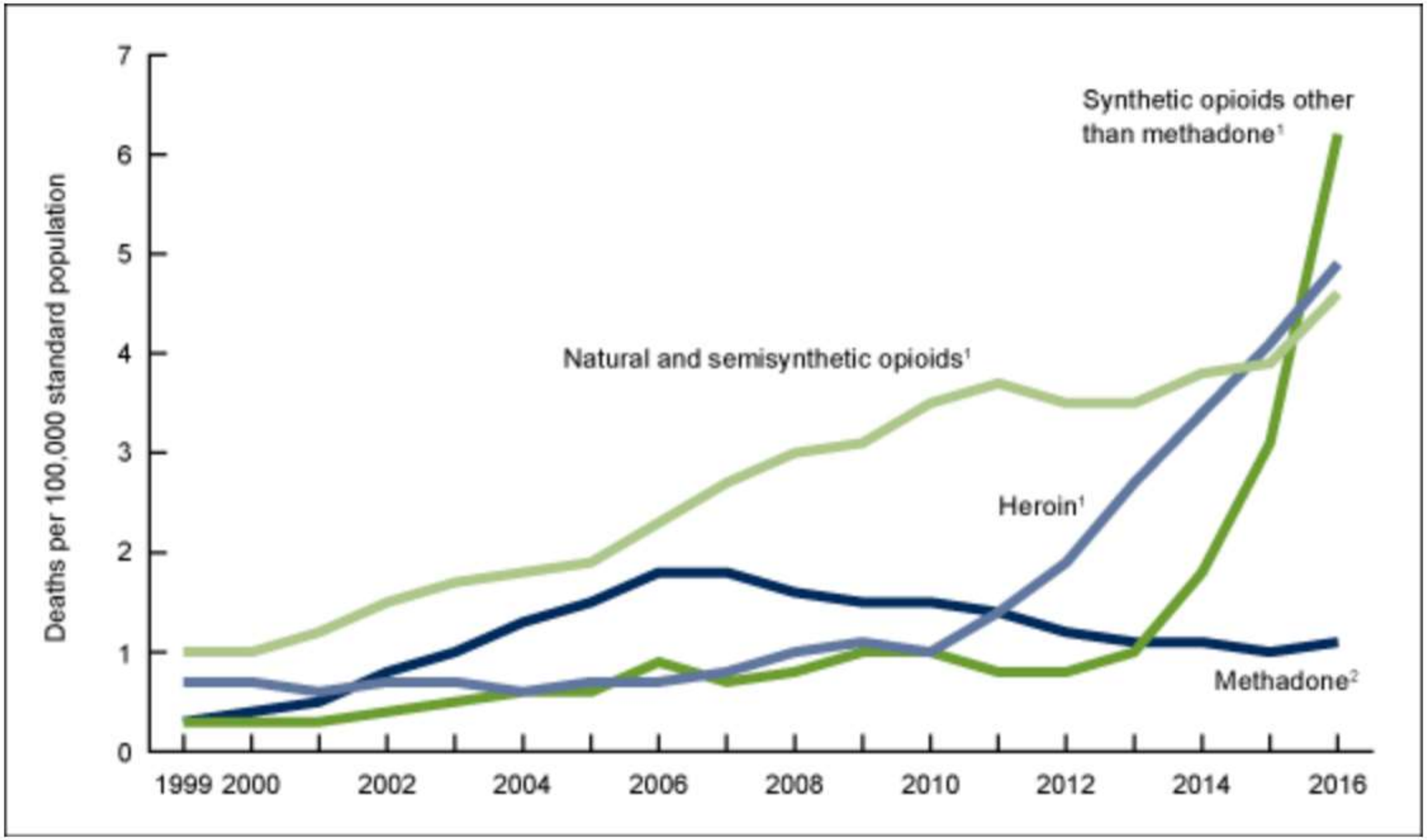

- NCHS Data Brief No. 294, December 2017

BRIGHAM HEALTH
BWH BRIGHAM AND WOMEN'S HOSPITAL

Fentanyl Epidemic

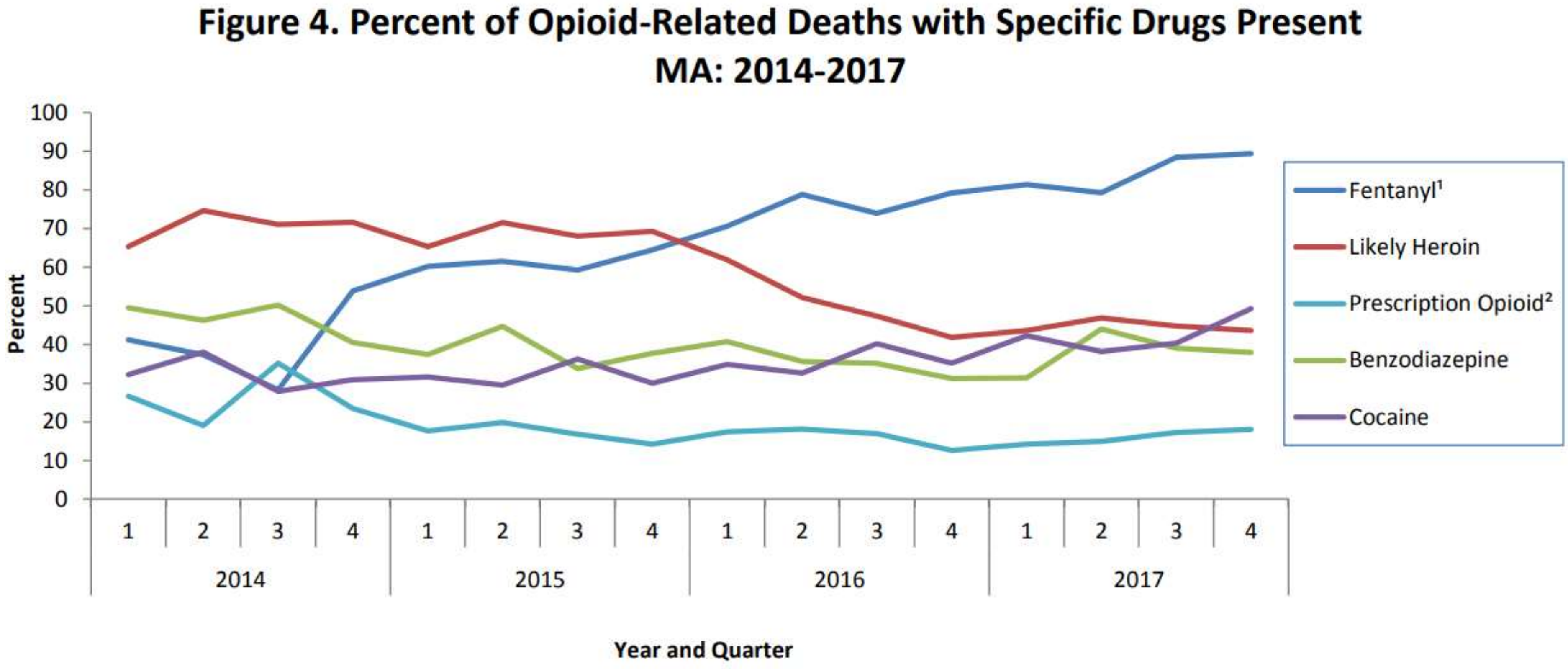

https://www.mass.gov/files/documents/2018/05/22/Opioid-related%20Overdose%20Deaths%20among%20MA%20Residents%20-%20May%202018.pdf

BRIGHAM HEALTH
BWH BRIGHAM AND WOMEN'S HOSPITAL

Fentanyl Epidemic

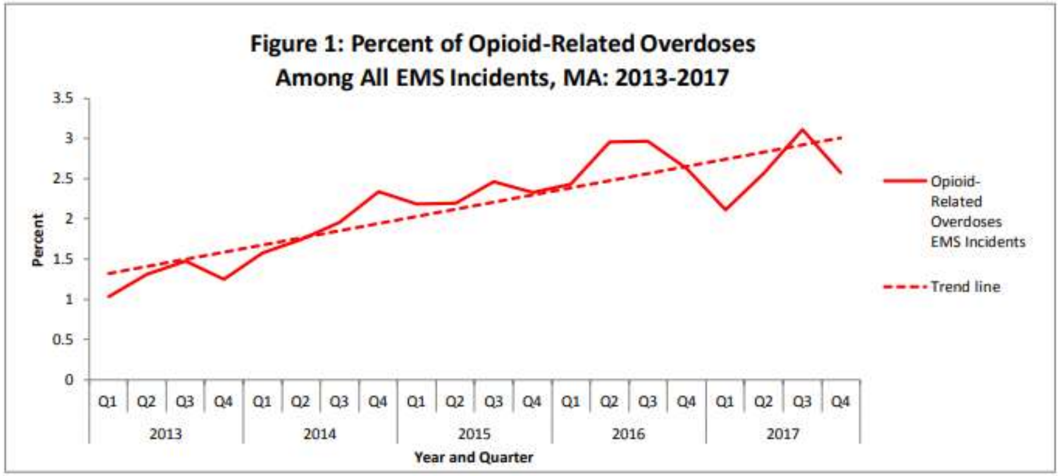

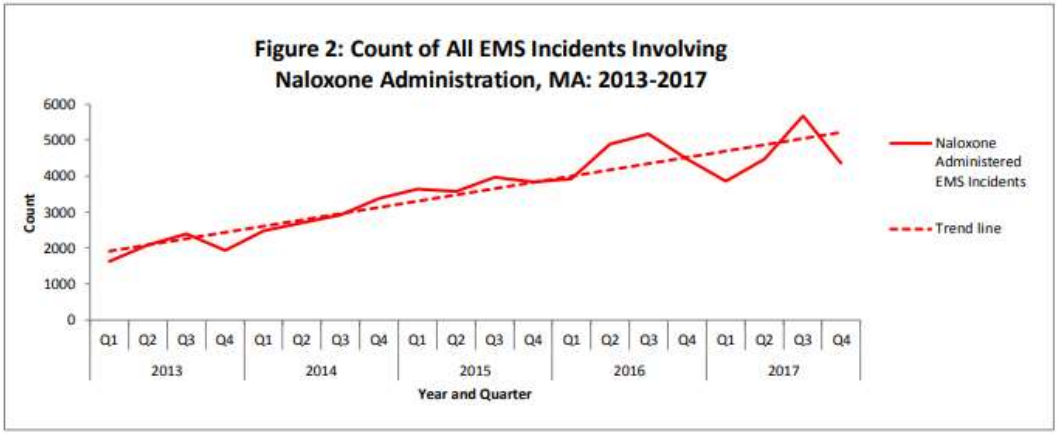

https://www.mass.gov/files/documents/2018/05/22/Opioid-related%20Overdose%20Deaths%20among%20
MA%20Residents%20-%20May%202018.pdf

Opiate vs. Opioid

- Opiates: alkaloids derived from the opium poppy
  - Morphine
  - Codeine
  - Heroin
  - Opium
- Opioids: synthetic or partly-synthetic drugs that are manufactured to work in a similar way to opiates.
  - Methadone
  - Percocet, Percodan, OxyContin (oxycodone)
  - Vicodin, Lorcet, Lortab (hydrocodone)
  - Demerol (pethidine)
  - Dilaudid (hydromorphone)
  - Duragesic (fentanyl)

Opioid Effects

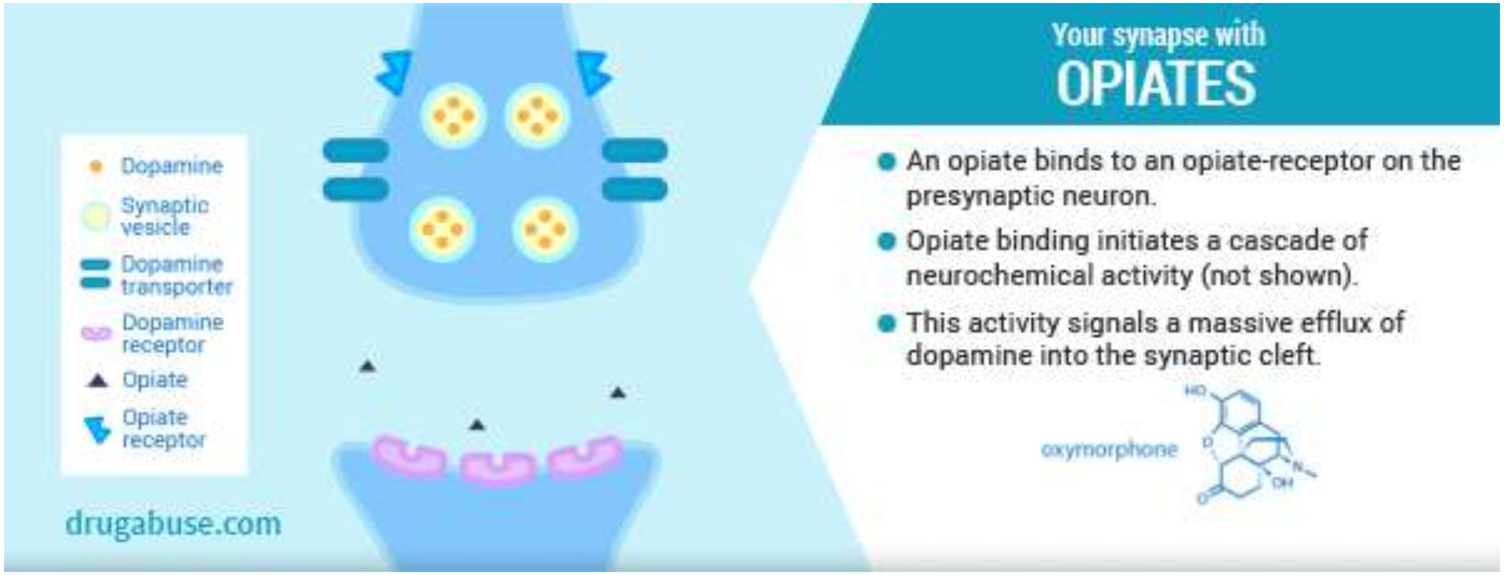

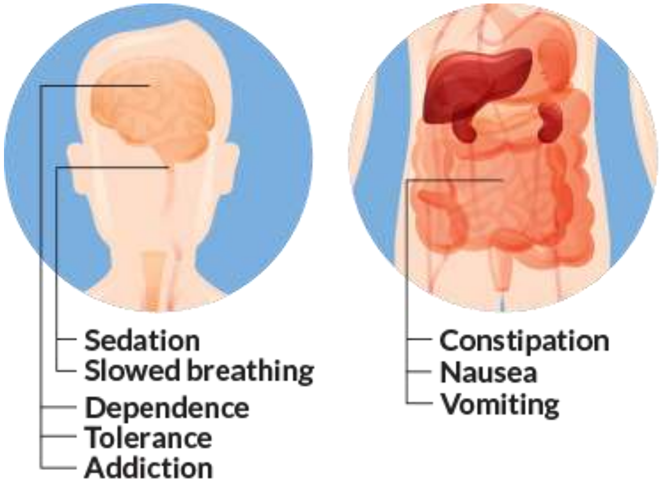

W

BRIGHAM HEALTH
BRIGHAM AND WOMEN'S HOSPITAL

Naloxone

- A medication called an “opioid antagonist” used to counter the effects of opioid overdose
- Counteracts life-threatening depression of the central nervous system and respiratory system, allowing an overdose victim to breathe normally.
- Naloxone is a nonscheduled (i.e., non-addictive), prescription medication.

Naloxone

- Naloxone only works if a person has opioids in their system; the medication has no effect if opioids are absent.
- Naloxone does not work for overdoses from other substances.
- Naloxone has no potential for abuse.
- Naloxone may be injected in the muscle, vein or under the skin or sprayed into the nose.
- It is a temporary drug that wears off in 20-90 minutes (typically about 60 minutes)

BRIGHAM HEALTH
BWH BRIGHAM AND WOMEN'S HOSPITAL

Naloxone

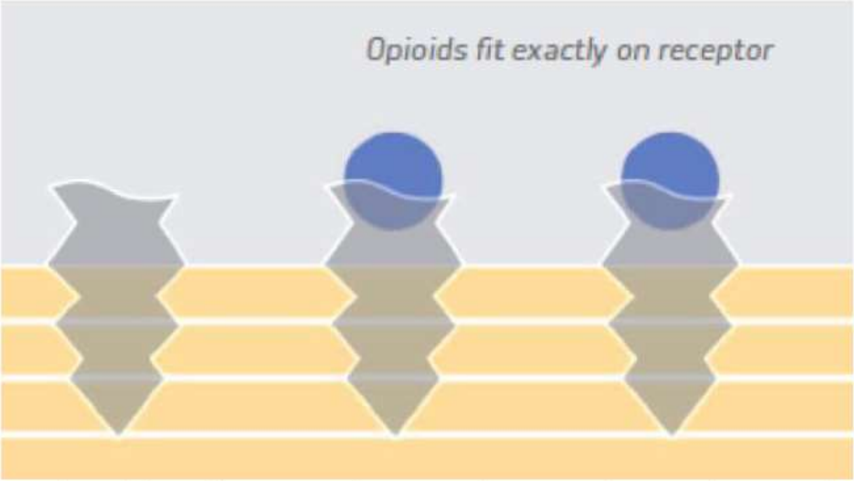

Adapted from the Harm Reduction Coalition: *Guide to Developing and Managing Overdose Prevention and Take-Home Naloxone Projects*.

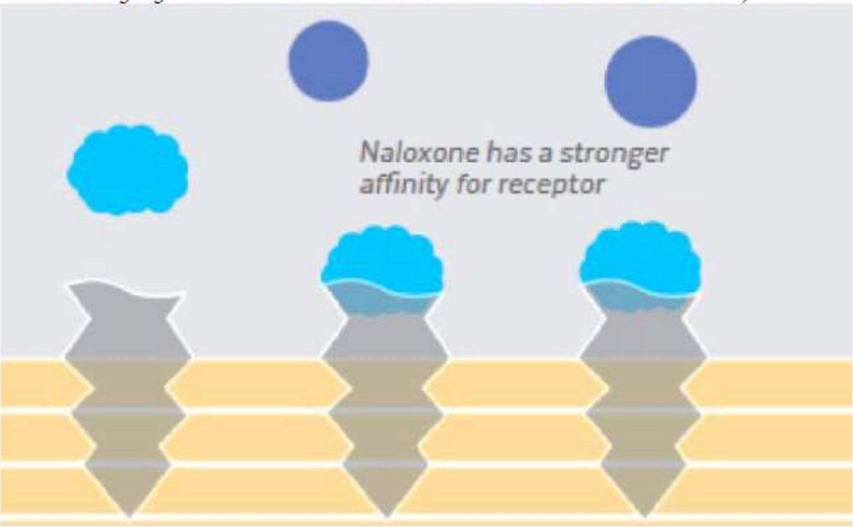

Risk Factors for Overdose

- Mixing opioids with other drugs, especially alcohol and benzodiazepines (Xanax®, Valium®, Ativan®)
- If a person hasn't been taking an opioid for an extended period of time and then starts taking it again, such as after being in
  - Jail
  - Detox program
  - Rehab treatment facility
- Using these medications while alone
- Fentanyl and other synthetics

Recognizing an Opioid Overdose

- If someone seems “high” but is still awake and able to walk/talk:
  - Get them up and walking around
  - Keep them talking to you

- Signs of overmedication:
  - Heavy nodding, sleepiness, but still responsive
  - Difficulty staying awake
  - Slurred or slow speech
  - Small pupils

Recognizing an Opioid Overdose

- Signs of Overdose:
  - Unresponsive to shouting, pain stimulation
  - Unconsciousness
  - Slow and shallow breathing or NOT breathing
  - Pale, clammy skin, loss of color
  - Blue, purple, or gray face, especially around lips/fingernails
  - Faint or NO pulse
  - Extremely small “pinpoint” pupils

Recognizing an Opioid Overdose

- What NOT to do during an overdose:
  - DO NOT put the individual into a cold bath or shower. They could drown.
  - DO NOT inject the person with any substance other than Naloxone (saltwater, milk, "speed", etc.)
  - DO NOT try to make the person vomit or give them something to eat or drink (e.g. coffee). They could choke.
  - DO NOT give over-the-counter drugs or vitamins (No-Doz, Niacin).

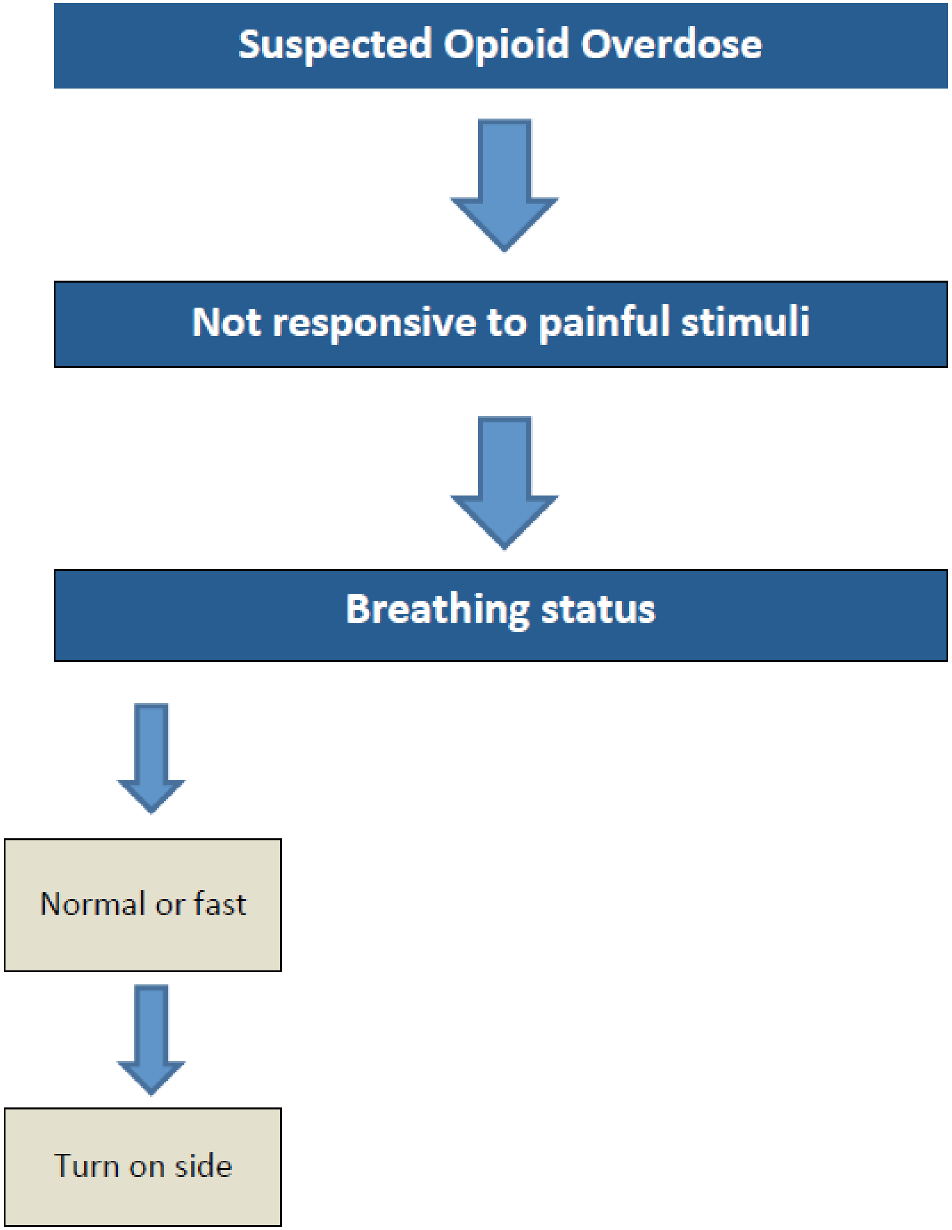

Courtesy of New York State Department of Health

BRIGHAM HEALTH
BWH BRIGHAM AND WOMEN'S HOSPITAL

Suspected Opioid Overdose

↓

Not responsive to painful stimuli

↓

Breathing status

Normal or fast	Slow – less than 10 x per minute	No or gasping
↓	↓	↓
Turn on side	Naloxone	Naloxone and CPR

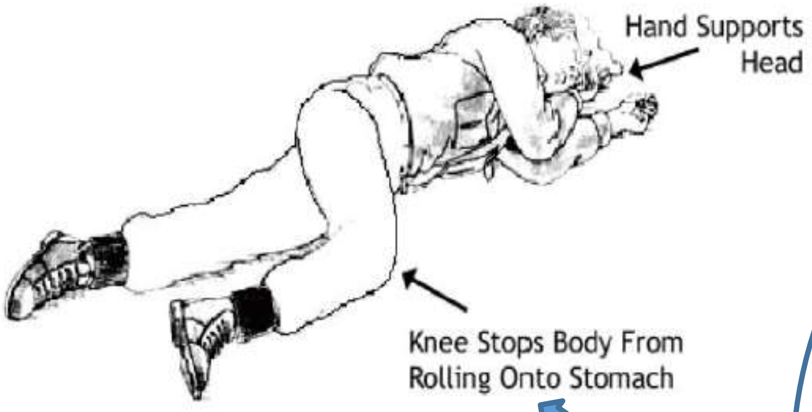

Courtesy of New York State Department of Health

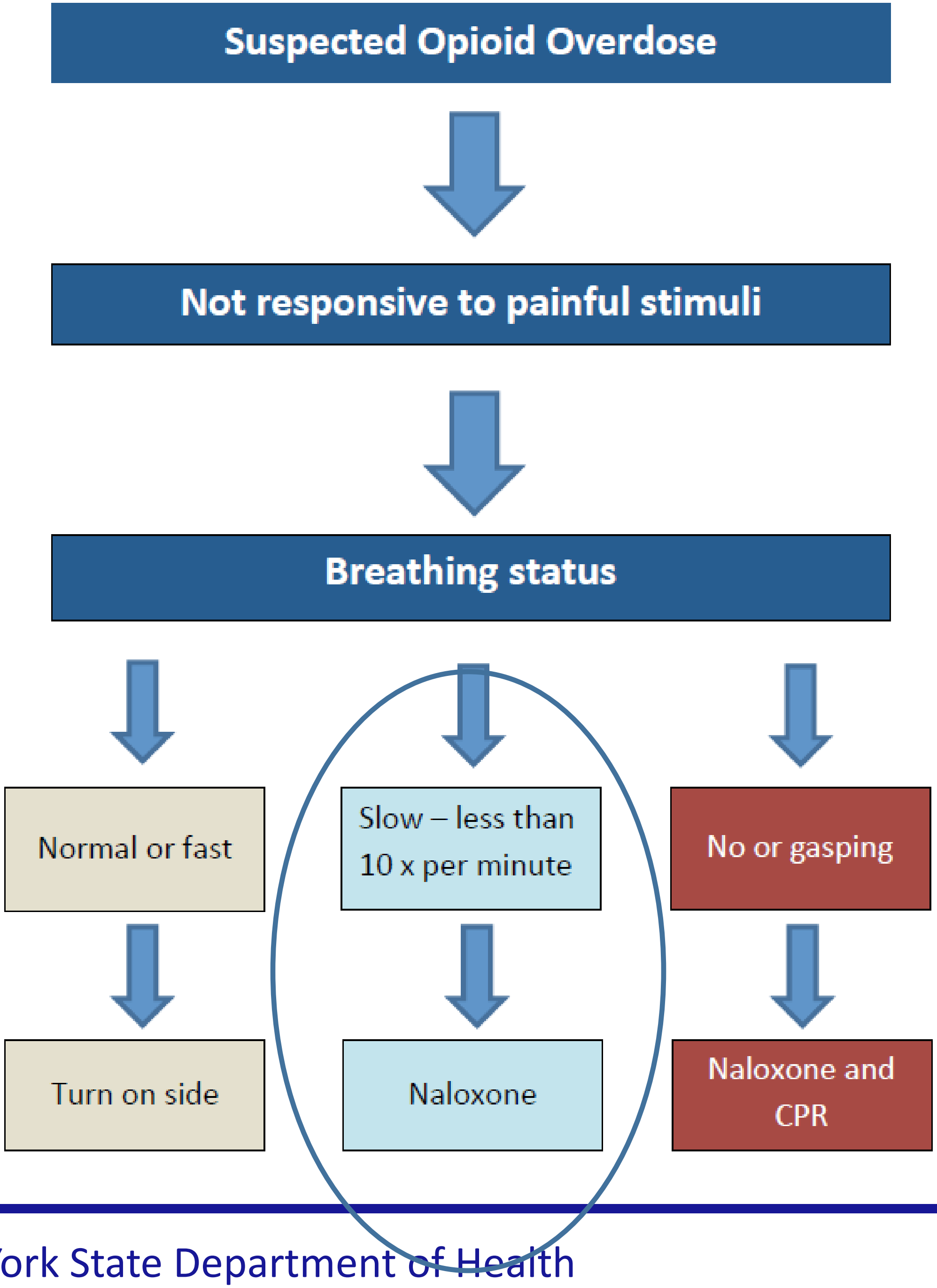

Courtesy of New York State Department of Health

BRIGHAM HEALTH
BRIGHAM AND WOMEN'S HOSPITAL

Naloxone

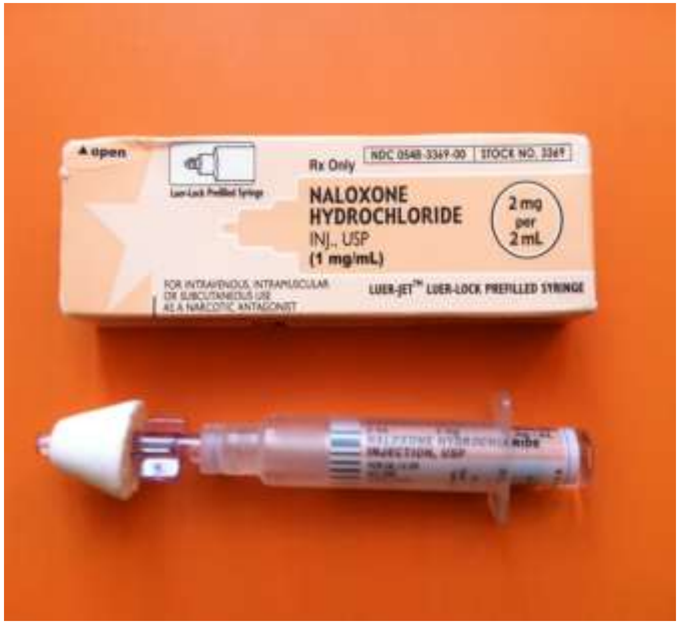

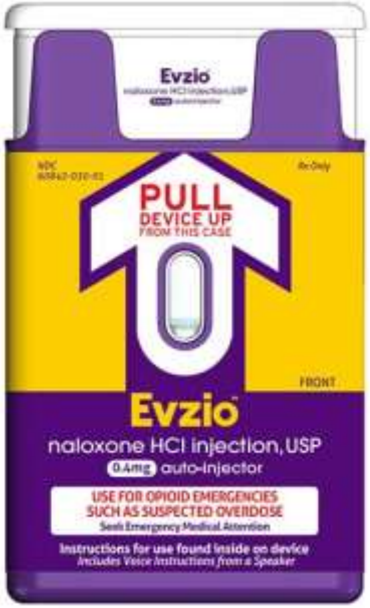

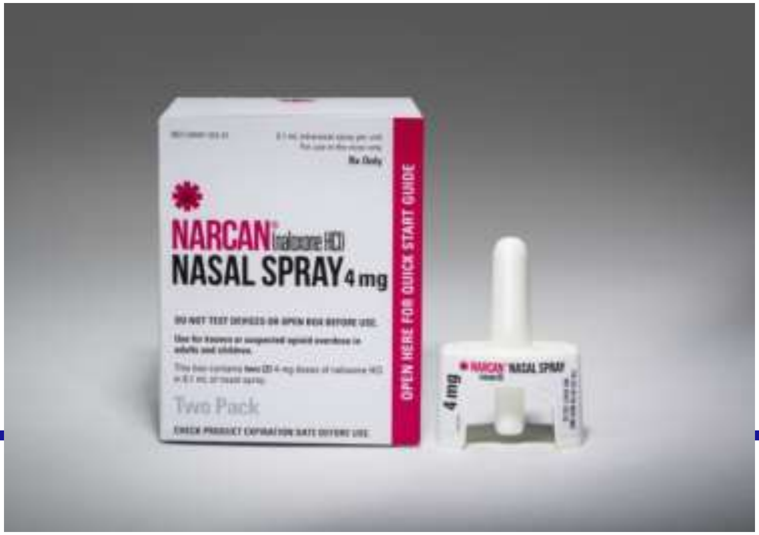

BRIGHAM HEALTH
BWH BRIGHAM AND WOMEN'S HOSPITAL

Naloxone

NARCAN® (naloxone HCl) NASAL SPRAY

QUICK START GUIDE

Opioid Overdose Response Instructions

Use NARCAN Nasal Spray (naloxone hydrochloride) for known or suspected opioid overdose in adults and children.
Important: For use in the nose only.
Do not remove or test the NARCAN Nasal Spray until ready to use.

1 Identify Opioid Overdose and Check for Response

Ask person if he or she is okay and shout name.

Shake shoulders and firmly rub the middle of their chest.

Check for signs of opioid overdose:

- Will not wake up or respond to your voice or touch
- Breathing is very slow, irregular, or has stopped
- Center part of their eye is very small, sometimes called "pinpoint pupils"

Lay the person on their back to receive a dose of NARCAN Nasal Spray.

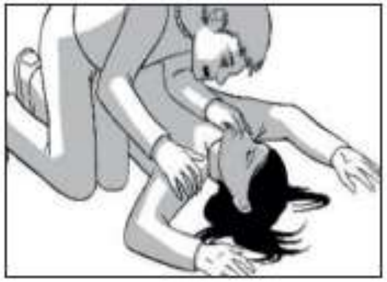

Courtesy of Adapt Pharma

Naloxone

2 Give NARCAN Nasal Spray

Remove NARCAN Nasal Spray from the box.
Peel back the tab with the circle to open the NARCAN Nasal Spray.

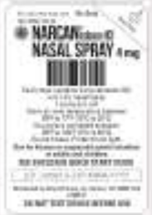

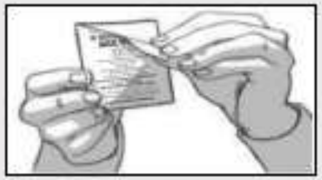

Hold the NARCAN nasal spray with your thumb on the bottom of the plunger and your first and middle fingers on either side of the nozzle.

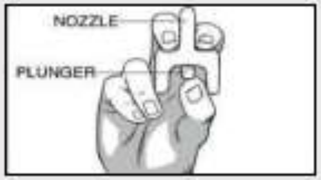

Gently insert the tip of the nozzle into either nostril.

- Tilt the person's head back and provide support under the neck with your hand. Gently insert the tip of the nozzle into **one nostril,** until your fingers on either side of the nozzle are against the bottom of the person's nose.

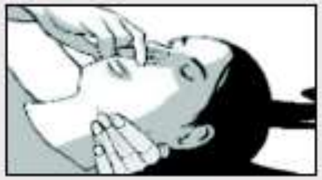

Press the plunger firmly to give the dose of NARCAN Nasal Spray.

- Remove the NARCAN Nasal Spray from the nostril after giving the dose.

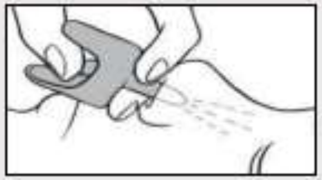

- Courtesy of Adapt Pharma

Naloxone

3 **Call for emergency medical help, Evaluate, and Support**

Get emergency medical help right away.

Move the person on their side (recovery position) after giving NARCAN Nasal Spray.

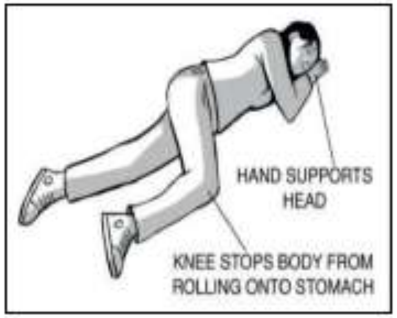

Watch the person closely.

If the person does not respond by waking up, to voice or touch, or breathing normally another dose may be given. NARCAN Nasal Spray may be dosed every 2 to 3 minutes, if available.

Repeat Step 2 using a new NARCAN Nasal Spray to give another dose in the other nostril. If additional NARCAN Nasal Sprays are available, repeat step 2 every 2 to 3 minutes until the person responds or emergency medical help is received.

- Courtesy of Adapt Pharma

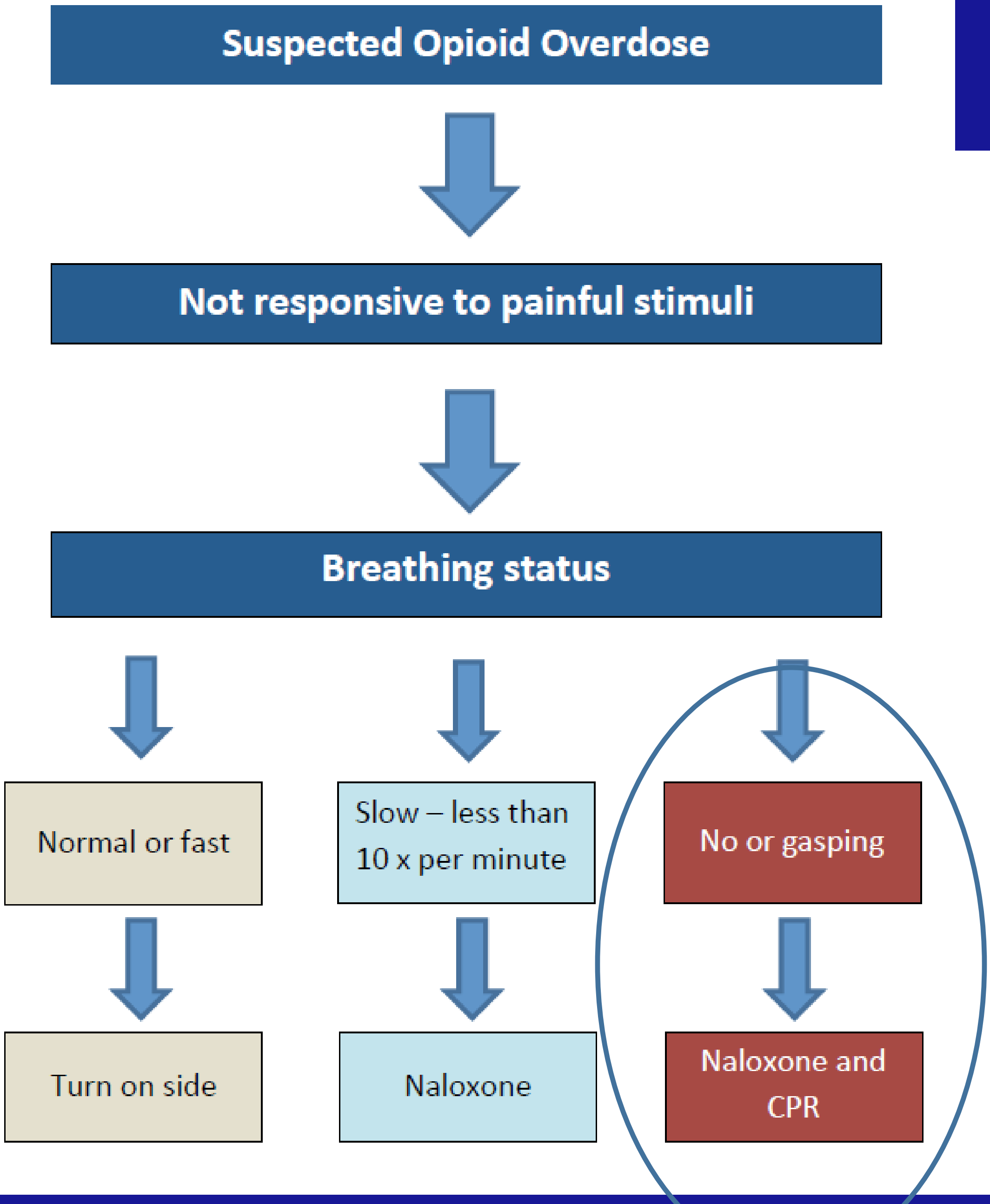

Courtesy of New York State Department of Health

BRIGHAM HEALTH
BRIGHAM AND WOMEN'S HOSPITAL

- If there is no response, CALL 911
  - Stay with person until emergency medical services arrive
  - Tell 911:
    - Address or location of where to find the person
    - If they are not breathing
    - If you gave Naloxone and how much
    - What medications the person took if you know
- Provide support to help blood circulation and oxygen delivery
  - Place one hand over the other on the person's sternum

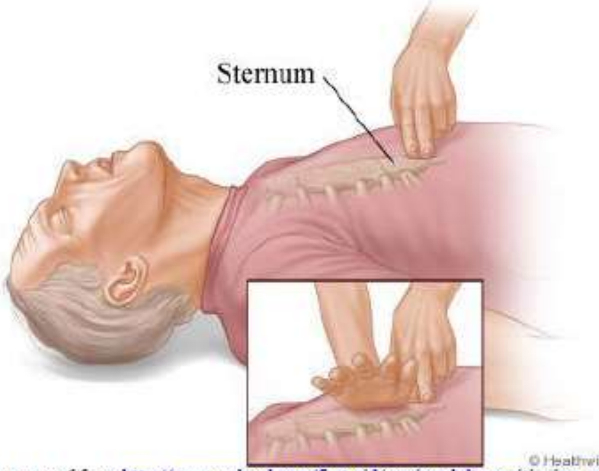

Retrieved Aug 23, 2016 from http://www.webmd.com/first-aid/cpr-in-adults-positioning-your-hands-for-chest-compressions

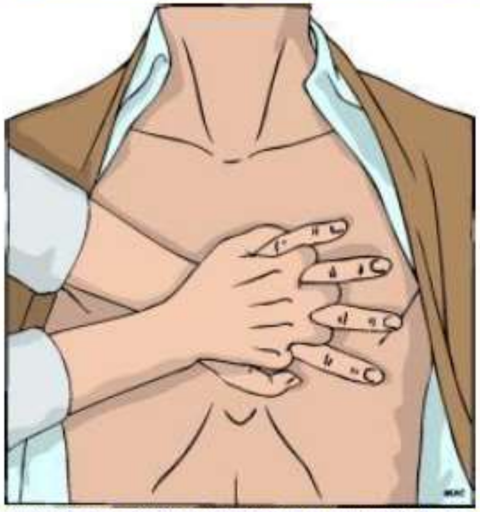

Retrieved Aug 23, 2016 from http://resuscitation-guidelines.articleinmotion.com/article/S0300-9572(10)00435-1/aim/adult-bls-sequence

Consider rescue breaths, 2 breaths after every 30 compressions

BRIGHAM HEALTH
BWH BRIGHAM AND WOMEN'S HOSPITAL

Evzio

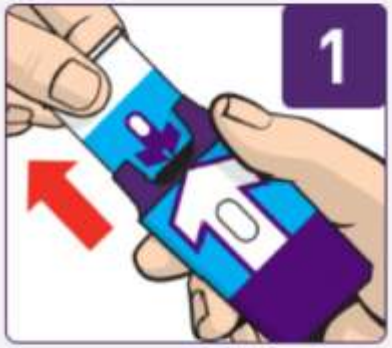

Pull EVZIO from the outer case.

Do not go to Step 2 (Do not remove the **red** safety guard) until you are ready to use EVZIO. **If you are not ready to use EVZIO, put it back in the outer case for later use.**

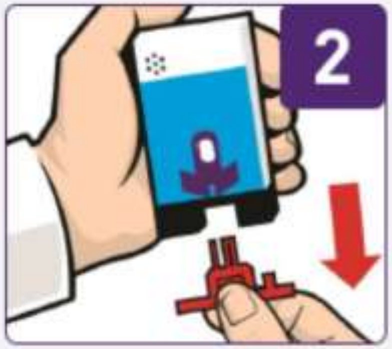

Pull off the **red** safety guard.

To reduce the chance of an accidental injection, do not touch the **black** base of the auto-injector, which is where the needle comes out. If an accidental injection happens, get medical help right away.

Note: The **red** safety guard is made to fit tightly. **Pull firmly to remove. Do not replace the red safety guard after it is removed.**

Evzio

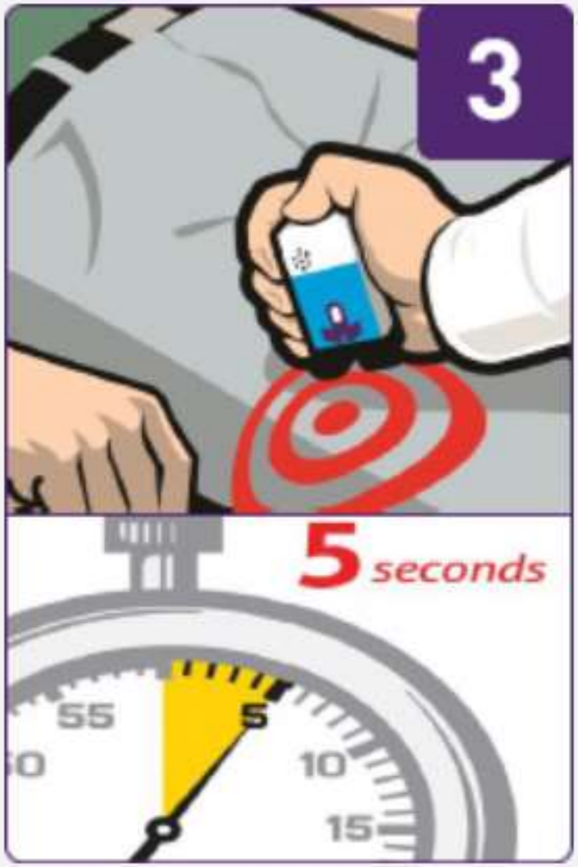

Place the **black** end of EVZIO against the outer thigh, through clothing, if needed. **Press firmly** and hold in place for 5 seconds. If you give EVZIO to an infant less than 1 year old, pinch the middle of the outer thigh before you give EVZIO and continue to pinch while you give EVZIO.

Note: EVZIO makes a distinct sound (click and hiss) when it is pressed against the thigh. This is normal and means that EVZIO is working correctly. Keep EVZIO firmly pressed on the thigh for 5 seconds after you hear the click and hiss sound. The needle will inject and then retract back up into the EVZIO auto-injector and is not visible after use.

Evzio

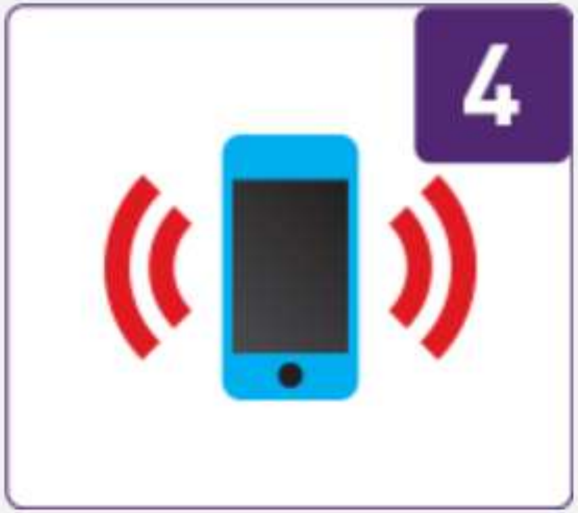

After using EVZIO, get emergency medical help right away. If symptoms return after an injection with EVZIO, an additional injection using another EVZIO may be needed. Give additional injections using a new EVZIO auto-injector every 2 to 3 minutes and continue to closely watch the person until emergency help is received. **EVZIO does not take the place of emergency medical care.**

EVZIO cannot be reused. After use, place the auto-injector back into its outer case. Do not replace the **red** safety guard.

Naloxone Nasal Atomizer

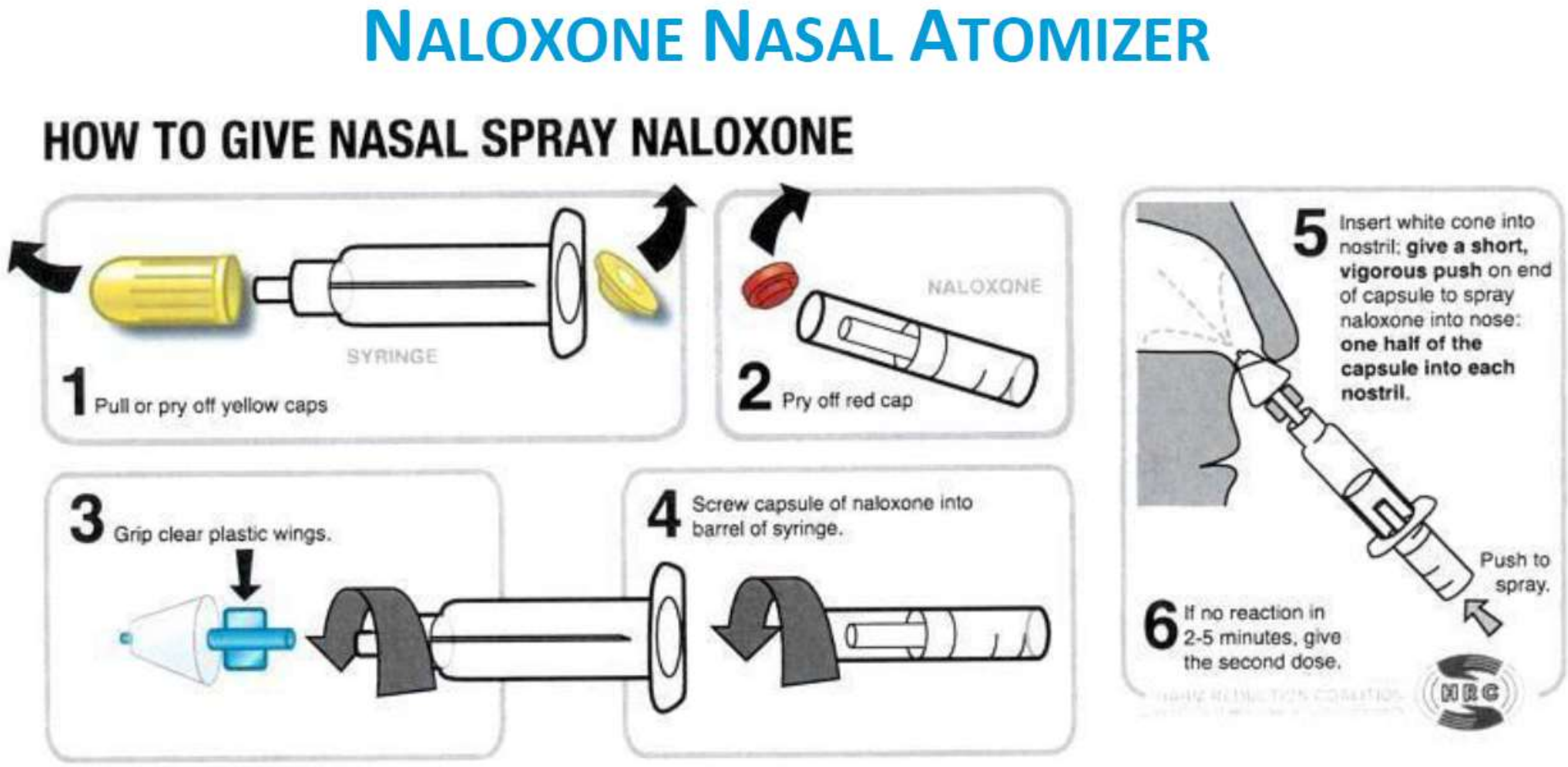

After Naloxone

- The person may have no memory of overdosing and you may have to explain that you've just given them Naloxone
- Comfort the person being treated, as withdrawal symptoms triggered by the Naloxone can feel unpleasant
- Help the person to remain calm
- Discourage the person from using more opioids for at least 2 hours
- Encourage the person to receive treatment from paramedics

After Naloxone

- Inform Paramedics:
  - When emergency medical services arrive tell them that Naloxone was given
  - If known, tell them what the person took and how much

BRIGHAM HEALTH
BRIGHAM AND WOMEN'S HOSPITAL

Good Samaritan

The law protects you.

The Massachusetts Good Samaritan Law encourages friends, family,or bystanders to assist people having an overdose and to seek emergency medical assistance. The law has significant potential to help reduce the impact of the opioid epidemic and save lives.

The law protects victims and those who call 9-1-1 for help from charge, prosecution, and conviction for possession or use of controlled substances. The Law, Chapter 94C, Section 34A: "Immunity from prosecution under Secs. 34 or 35 for persons seeking medical assistance for self or other experiencing a drug-related overdose" can be found on the Massachusetts Legislature General Laws website.

- (e) A person acting in good faith may receive a naloxone prescription, possess naloxone and administer naloxone to an individual appearing to experience an opiate-related overdose.

Summary

RESPONDING TO AN OPIOID OVERDOSE	
☐ CONFIRM UNRESPONSIVE	• **Stimulate** with: **Noise** (shout, use their name), **Pain** (ex. sternal rub) – Remember, tell person what you are doing before you touch them
☐ CALL 911 or local emergency response number	• Put person in the recovery position if you have to leave them alone. The Good Samaritan Drug Overdose Act protects the caller from drug possession charges
☐ CLEAR AIRWAY	• Clear airway (is there anything in their mouth?), tilt head, lift chin<br>• Pinch nose and give 2 breaths. If no response: administer naloxone if you have it
☐ GIVE INTRAMUSCULAR NALOXONE *(demonstrate if possible)*	• Swirl ampoule, snap top off, draw up all of the naloxone, remove most of the excess air<br>• Inject into large <u>muscle</u> – **THIGH**, or upper arm<br>• Inject at 90°, push plunger until you hear a click (needle will retract)
☐ GIVE BREATHS AND CHEST COMPRESSIONS	• If the person is barely breathing or not breathing: commence cycles of 30 chest compressions to 2 rescue breaths<br>• 911 will review these instructions
☐ EVALUATE EFFECTS (for 4-5 minutes) & GIVE MORE NALOXONE IF NEEDED	• **Continue to give breaths <u>FOR 3-5 MINUTES</u> (about 7 CPR cycles)** OR until they respond (are breathing again on their own).<br>• **<u>After 3- 5 minutes, if still unresponsive, give a 2nd dose of naloxone</u>**<br>• **Continue CPR and naloxone administration every 3-5 minutes** until person breathing OR paramedics arrive OR you run out of naloxone (continue CPR only)
☐ AFTERCARE AND CARING FOR NALOXONE	• Naloxone wears off in 20-90 minutes<br>• Person will not remember overdosing – explain what happened<br>• If person does NOT go to hospital monitor at least 2 hours and do NOT allow them to take more opioids (could OD again)Naloxone should be stored out of the light at room temperature (15-30 C)<br>• Be aware of the expiry date – it is on the ampoule

FAQs

Q&A
